# The Influence of Volunteers’ Psychological Capital: Mediating Role of Organizational Commitment, and Joint Moderating Effect of Role Identification and Perceived Social Support

**DOI:** 10.3389/fpsyg.2020.00673

**Published:** 2020-04-22

**Authors:** Li ping Xu, Yu shen Wu, Jing jing Yu, Jie Zhou

**Affiliations:** ^1^Department of Social Science, Zhuhai Campus of Zunyi Medical University, Zhuhai, China; ^2^Faculty of Psychology, Beijing Normal University, Beijing, China; ^3^Department of Police Management, Sichuan Police College, Luzhou, China

**Keywords:** volunteering, psychological capital, organizational commitment, role identification, perceived social support

## Abstract

This study explores the relationship between volunteers’ psychological capital and their commitment to volunteering. We tested whether volunteers’ psychological capital had a positive predictive effect on volunteering and whether this effect was mediated by organizational commitment, role identification, or perceived social support. A sample of 1165 volunteers who were registered in the national volunteer service information system of China were recruited in the study. The results showed a significant and positive relationship between volunteers’ psychological capital, volunteering, role identification, perceived social support, and organizational commitment. Volunteers’ psychological capital not only had a direct effect on volunteering but also affected volunteering through the mediating role of organizational commitment. Additionally, the influence of the volunteers’ psychological capital on organizational commitment was affected by the joint moderated effect of role identification and perceived social support. Volunteers with low role identification and low perceived social support, high role identification and low perceived social support, and low role identification and high perceived social support committed to their volunteer organization faster when they had a high level of psychological capital; whereas, volunteers with high role identification and high perceived social support committed to their volunteer organization faster when they had a low level of psychological capital.

## Introduction

Volunteers are organized actors who voluntarily provide public service without making any profit or fame; thus, the scale, service effect and sustainability of volunteer work greatly affects the health condition of civil society (Musick and Wilson, 2003; Hustinx et al., 2010). The Chinese government and the Communist Party of China (CPC) have been paying close attention to voluntary service activities. For example, the 19th National Congress of CPC proposes that we should promote the construction of good faith, the institutionalization of voluntary service, and the strengthening of the consciousness of social responsibility, rules and dedication. Furthermore, while inspecting Tianjin in January 2019, President Xi Jinping emphasized the value of voluntary service and its meaningful relationship to the Two Centenary Goals. Volunteers make important contributions to large-scale competitions, emergency rescue, and community service at home and abroad; their work helps to make up for the lack of governmental and market services and is beneficial to the development of social harmony.

The effectiveness of voluntary service is closely related to the psychological state of volunteers (Min and Ming-Jie, 2017). Therefore, in order to strengthen and promote voluntary service, it is necessary to continue researching ways to improve the psychological wellbeing of volunteers. However, previous studies on volunteers focused on volunteer motivation and function (Arbak and Villeval, 2013; Dickson et al., 2013, 2015; Erasmus and Morey, 2016; Li et al., 2016), voluntary incentive and management (Prestby et al., 1990; Edwards, 2007; Dickson et al., 2013; Gong, 2018; King, 2018), volunteerism and values (Beverley, 1975; Johnson et al., 1998; Misener et al., 2009; Shen et al., 2019), and leisure perspective (Stebbins, 1996; Green and Chalip, 2004). Only a few studies have looked at psychological mechanisms that influence volunteering (Spector and Fox, 2002; Kang et al., 2007; Fornyth, 2010; Chacón et al., 2017). Moreover, previous research on volunteering has mostly focused on cultural capital (Harflett, 2015), social capital (Bailey et al., 2003; Wang and Graddy, 2008), and human capital (Choi and Chou, 2010; Lindsay, 2016) at home and abroad; yet research on psychological capital with respect to volunteering has yet to appear.

Psychological capital–which surpasses social capital, cultural capital, and human capital–is an inexhaustible force and has a more significant impact on individual attitude and behavior (Luthans et al., 2007). However, the exact relationship between psychological capital and volunteering remains to be further explored. Therefore, our study sought to understand how psychological capital influences volunteering in order to provide a new theoretical perspective, enrich the application of psychological capital in volunteers, and widen the research on the sustainable development of volunteering.

## Theory and Hypothesis

### The Positive Predictive Effect of Volunteers’ Psychological Capital on Volunteering

Psychological capital is a kind of positive mental state or psychological quality that individuals possess throughout the process of growth and development; it includes four dimensions: self-efficacy, optimism, resilience, and hope (Luthans et al., 2004). According to the resource conservation theory, psychological capital is also a kind of psychological resource which helps to strengthen the emotional connection between individuals and organizations (Hobfoll et al., 2018). It has been found that the impact of psychological capital on the attitude and behaviors of individuals is strong (Luthans et al., 2007; Avey et al., 2011), and surpasses material capital, human capital, and social capital (Luthans et al., 2007). Studies have found that high levels of psychological capital and volunteering are associated with increased odds of older adults using the internet for health-related tasks (Choi and DiNitto, 2013). Individuals who are more confident/efficacy, hopeful, resistant to setbacks, and inclined toward optimism show more altruistic behavior (Myers, 2012; Min and Ming-Jie, 2017). Since volunteering is an altruistic behavior, the present study hypothesized that volunteers’ psychological capital would positively predict volunteering (H1).

### The Mediating Effect of Volunteers’ Organizational Commitment

Organizational commitment has been defined as a psychological index measuring the relation between a volunteer and the quality of the relationship they have with their volunteer organization (Meyer et al., 1993). Studies have shown that individuals’ psychological capital can effectively predict organizational commitment and values (Green and Chalip, 2004; Larson and Luthans, 2006; Shahnawaz and Jafri, 2009), and individuals’ organizational commitment and values has a positive prediction on their attitude and behavior (Spector and Fox, 2002; Cooper-Hakim and Viswesvaran, 2005; Edwards, 2007; Matsuba et al., 2007). At present, the high draining rate of volunteering and mental instability of volunteers greatly impact the formation of positive working atmospheres and seriously hinder the sustainable development of volunteering. To face this challenge, it is necessary for volunteer organizations to cultivate the organizational commitment of volunteers in various ways to strengthen the volunteers’ psychological attachment and emotional connection with their respective organizations. Studies show that the first six months after individuals enter an organization is usually the critical period of developing their role identification and organizational commitment (Kramer, 1994). Therefore, the present study also hypothesized that organizational commitment would play a mediating role in the impact volunteers’ psychological capital has on volunteering (H2).

### The Joint Moderated Effect of Role Identification and Perceived Social Support

Role identification is the internalization or self-definition of the role expectation that individuals possess (Stryker and Burke, 2000). Role identification is also a significant source of self-concept and the individuals’ self-image of being at a certain social level (Mccall and Simmons, 1966) and is closely related to donation and volunteering (Penner and Finkelstein, 1998; Lee and Lee, 1999; Grube and Piliavin, 2000; Finkelstein et al., 2005; Finkelstein, 2008). Resource conservation theory considers role identification to be another psychological resource that increases volunteering behavior. Role identification theory posits that self-concept and social relations are two important sources of self-identification (Riley and Burke, 1995). According to resource conservation theory (Hobfoll et al., 2018), role identification is also an important psychological resource that increases organizational commitment.

Volunteers’ role identification can change with the change of factors such as social expectation and feedback from other volunteers, self-evaluation and awakening of volunteers, and the experiences of success and failure (Callero et al., 1987; Riley and Burke, 1995). At the same time, role identification of volunteers with a background in Chinese culture usually presents aspects such as value identification, emotional connection, and the loyal devotion of individuals to their volunteer organization; the level of this devotion will lead to a change in organizational commitment (Edwards, 2005). Therefore, the effect of role identification on the relation between volunteers’ psychological capital and organizational commitment can be speculated. In addition, both trait activation theory and resource conservation theory consider that the behavior and attitude of individuals are influenced by their internal psychological resources and external situation such that we cannot examine the internal psychological factors isolated from the external situation (Tett and Guterman, 2000). The moderated effect of role identification cannot effectively work unless in a specific situation.

One type of external situation is perceived social support, which refers to the various levels of social support which individuals can perceive from friends, family, and others. Perceived social support has an important role in activating the level of role identification and regulating the relationship between psychological capital and volunteering. According to the social exchange theory, there is an exchange relationship between people; when individuals receive help and support from others, they tend to pay it forward in return (Wilson et al., 2010). Studies have shown that supportive feedback environment helps to improve employe role clarity, job satisfaction and performance, as well as the professional adaptability of nurses (Gong and Li, 2019). Volunteers view support from their organization, family, and friends as a kind of potential pressure and driving force of development, and as such, they try their best to respond with their own altruistic actions. Studies find that a high level of perceived social support (Conn et al., 2004; Kim and Hopkins, 2015) and role identification (Kumar et al., 2012) have a positive predictive effect on improving organizational commitment (Griffin et al., 2000) and increasing volunteering (Piliavin and Callero, 1991). Based on these findings, the present study hypothesized the following: that role identification would play a moderating role on the impact of volunteers’ psychological capital on organizational commitment (H3), and that role identification and perceived social support would play a joint moderating role on the impact of volunteers’ psychological capital on organizational commitment (H4).

### The Present Study

**FIGURE 1:**
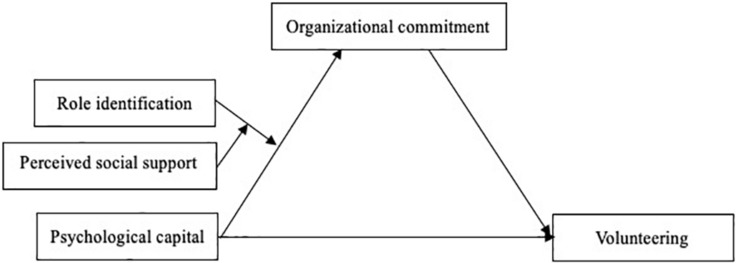
The mechanism effect of volunteers’ psychological capital on volunteering.

The present study focused on the relation between volunteers’ psychological capital and volunteering, as well as the mediating effect of organizational commitment and the joint moderating effects of role identification and perceived social support. Based on the above theory and hypothesis of this study, a theoretical model of the mechanism effect of volunteers’ psychological capital on volunteering was constructed in Figure 1.

## Materials and Methods

### Participants and Procedure

The present study was approved by seven universities (including South China University of Technology, Campus of Zhuhai of Zunyi Medical University, Southern Medical University, Linyi University, Guangdong Polytechnic of Industry and Commerce, Yichun University, and the Guangdong Teachers College of Foreign Language and Arts) and six social workers’ organizations located throughout the Guangdong, Shandong, Guizhou, and Jiangxi provinces, as well as in Shanghai, China. We contacted the leaders of the voluntary organization within each unit and, with their help, randomly distributed about 100–200 questionnaires (1600 questionnaires in total). After giving their informed consent, the participants were instructed to complete the survey on-site; their data was kept completely anonymous.

We recovered 1204 questionnaires. After eliminating 39 questionnaires with incomplete information, we obtained 1165 valid questionnaires with a questionnaire efficiency of 96.8%. The inclusion criteria for the participants was an active registration in the China voluntary service information system. The participant pool consisted of 39.6% males (461) and 60.4% females (704), and age from 16 to 68. There were 51.8% college students (603), 3.4% civil servants (40), 20.5% personnel of enterprises and institutions (239), 16.7% freelancers (195), and 7.6% retirees (88). In terms of service duration, 42.0% had been volunteering for less than one year (489), 35.0% between 1 and 3 years (408), 14.8% between 3 and 5 years (172), and 8.2% more than 5 years (96).

### Measures

#### Psychological Capital

This questionnaire was compiled by Chinese scholar Kuo et al. (2010). In accordance with the theory of Luthans et al. (2007), the questionnaire included the four dimensions of psychological capital: self-efficacy, optimism, hope, and resilience. The questionnaire consists of 26 items and uses a 7-point Likert scale. Self-efficacy refers to an individual’s self-confidence about his or her ability to face challenges, complete the required tasks, and strive for success. This dimension contains 7 items, such as “I am happy to take on difficult and challenging work (1 = strongly agree, 7 = strongly disagree)”. Optimism refers to a positive attitude and outlook regarding the present and future. This dimension contains 6 items, such as “I always see the good side of things (1 = strongly agree, 7 = strongly disagree)”. Hope refers to the state of positive motivation to achieve the intended goal through various channels. This dimension contains 6 items, such as “I pursue my goals with confidence (1 = strongly agree, 7 = strongly disagree)”. Resilience refers to the ability to recover quickly from adversity, frustration, and failure. This dimension contains 7 items, such as “I can quickly recover from frustration (1 = strongly agree, 7 = strongly disagree)”. We calculated an average score for each item; the higher the score for each dimension, the higher the level of each factor. This scale was reliable in this study (Cronbach’s α = 0.991).

#### Perceived Social Support

We used the perceived social support questionnaire to assess various levels of social support that individuals perceived from friends, family, and others. This questionnaire was developed by Blumenthal et al. (1987) and has been translated into Chinese by Zhong et al. (2006). The questionnaire used a 7-point Likert scale and consisted of 12 items such as, “There is a special person who is around when I am in need (1 = very strongly disagree, 7 = very strongly agree)”. We calculated an average score for each item. The higher score for each dimension means that the individual perceives a higher level of social support. This scale was reliable in this study (Cronbach’s α = 0.897).

#### Volunteering

The present study used the volunteering questionnaire developed by Carlo et al. (2005). This questionnaire consisted of 4 items, Volunteers were asked whether they had ever volunteered (no = 0, yes = 1), were currently volunteering (no = 0, yes = 1), planned on volunteering during the next two months (no = 0, yes = 1), and the likelihood that they would volunteer at the campus-based community service program if asked (definitely no = 0, probably no = 1, may be = 2, probably yes = 3, and definitely yes = 4). The total score of this questionnaire ranged from 0 to 7. We calculated an average score for each item by dividing the total score by 4 items. The higher the average score, the more likely individuals are to participate in volunteering. This scale was reliable in this study (Cronbach’s α = 0.762).

#### Organizational Commitment

This scale used a revised version of the organizational commitment scale developed by Meyer et al. (1993), and included three dimensions: affective commitment, normative commitment, and continuance commitment. The questionnaire consisted of 18 items and used a 5-point Likert scale. Affective commitment refers to the emotional attachment and identification with the organization. This dimension contains 6 items, such as “I have a strong sense of belonging to the organization (1 = strongly agree, 5 = strongly disagree)”. Normative commitment refers to the individual’s personal commitment to stay in the organization. This dimension contains 6 items, such as “I am willing to do my best to cooperate with various institutional measures in the organization (1 = strongly agree, 5 = strongly disagree)” Continuance commitment refers to the willingness to remain in the organization based on utilitarian considerations. This dimension contains 6 items, such as “I will continue and stay in the organization for a long time (1 = strongly agree, 5 = strongly disagree).” We calculated an average score for each item; the higher the average score, the higher the level of organizational commitment. This scale was reliable in this study (Cronbach’s α = 0.917).

#### Role Identification

This scale revised the item statement of the job role identification scale compiled by Saleh and Hosek (1976), for example, replacing the phrase “the job” with “the voluntary service job.” The scale consisted of 4 items, such as, “I’m very dedicated to my current volunteer role”. All items were rated on a 5-point scale, ranging from 1 (strongly agree) to 5 (strongly disagree). We calculated an average score for each item; the higher the average score, the stronger individuals self-identified with their role within a voluntary service. The scale was reliable in this study (Cronbach’s α = 0.745).

#### Data Analysis

The study used SPSS 24.0 to carry out descriptive statistical and correlation analyses on the 1165 questionnaires and adopted the Bootstrap inspection of the PROCESS procedure for SPSS 24.0 (Hayes, 2013). This software was used to investigate the mediating effect of organizational commitment, the moderating effect of role identification, and perceived social support in relation to volunteers’ psychological capital and organizational commitment.

## Results

### Common Method Deviation Analysis

The study adopted Haman single factor analysis to carry out the common method deviation analysis on all the valid data. As a result, the present study found that there were 17 factors featuring root values greater than one and that the variance of the first one was 27.322%, smaller than the critical value of 40%. The present study also carried out the confirmatory analysis of the single factor model, and results showed that the model was a poor fit (^χ^2/df = 21.13, CFI = 0.46, TLI = 0.45, RMSEA = 0.13), which meant that the common deviation method of the study was not remarkable.

### Preliminary Analysis

The results of descriptive statistics and correlation analysis are shown in Table 1. Results revealed that volunteers’ psychological capital had a positive correlation with factors such as volunteering, organizational commitment, and role identification. The correlation coefficient was 0.281–0.652 (*p* < 0.01), which showed that it was necessary to further reveal the internal relationship between the elements.

**TABLE 1:** Correlation coefficients, means, and standard deviations of variables.

	M	SD	1	2	3	4
1. Psychological Capital	5.080	0.749	——			
2. Volunteering	1.378	0.412	0.400**	——		
3. Organizational Commitment	4.034	0.557	0.521**	0.375**	——	
4. Role Identification	4.045	0.619	0.465**	0.346**	0.652**	——
5. Perceived Social Support	5.586	0.925	0.495**	0.281**	0.522**	0.451**

*Represents *p* < 0.05; **represents *p* < 0.01.

### Hypothesis Testing

Studies at home and abroad showed that gender is an important factor impacting voluntary service behavior (Haski-Leventhal et al., 2008); it is widely believed that women are more likely to participate in volunteering than men (Moore et al., 2014). Therefore, the present study viewed gender as the control variable when analyzing the mediating model of joint regulation in the relationship between volunteers’ psychological capital and volunteering activity. Besides, the study carried out centralized treatment on the variable data to avoid the multicollinearity between the variables. On this basis, the present study adopted the model 11 of the PROCESS procedure (this model assumed that the aggregate variable regulated the first half path of the mediation model, in accordance with the theoretical model of this study) to carry out the Bootstrap inspection of the moderated mediating model, setting the self-sampling number to 5000.

The result of joint moderated mediation effect showed that the volunteers’ psychological capital had a significant total effect on the prediction of volunteering (β = 0.26, *p* < 0.01). Therefore, Hypothesis 1 was true: Volunteers’ psychological capital had a positive predictive effect on both volunteering and organizational commitment after putting all the study factors into the regression equation (see Table 2). The significance of the mediating effect also indicated a mediating effect of organizational commitment. The Bootstrap 95% confidence intervals did not include 0, which meant that the volunteers’ psychological capital had a positive effect on volunteering through the mediating effect of organizational commitment. Therefore, Hypothesis 2 was also true.

**TABLE 2:** Joint moderated mediation effect analysis.

Regression equation		Overall fit index			Significance in regression coefficient			
Result Variable	Predictive variables	R	R2	F	ß	LLCI	ULCI	t
Volunteering		0.40	0.16	111.54**				
	Gender				0.030	–0.019	0.070	1.130
	Psychological Capital				0.403	0.192	0.250	14.936**
		0.73	0.53	162.07**				
Organizational Commitment	Gender				0.045	0.001	0.091	1.958
	Psychological Capital				–1.132	–2.001	–0.265	−2.562*
	Role Identification				–1.520	–2.524	–0.516	−2.967**
	Psychological Capital × Role Identification				0.363	0.146	0.580	3.282**
	Perceived Social Support				–1.384	–2.144	–0.625	−3.579**
	Psychological Capital × Perceived Social Support				0.280	0.120	0.440	3.441**
	Role Identification × Perceived Social Support				0.411	0.223	0.600	4.274**
	Psychological Capital × Role Identification × Perceived Social Support				–0.077	–0.116	–0.037	−3.843**
Volunteering		0.45	0.20	95.66**				
	Gender				0.009	–0.035	0.053	0.394
	Organizational Commitment				0.168	0.123	0.213	7.334**
	Psychological Capital				0.155	0.122	0.189	9.107**

*represents *p* < 0.05; **represents *p* < 0.01.

In addition, the product of the volunteers’ psychological capital and role identification (β = 0.363, *p* < 0.05), volunteers’ psychological capital and perceived social support (β = 0.411, *p* < 0.01), and volunteers’ role identification and perceived social support (β = 0.411, *p* < 0.01) all had remarkable positive predictive power of organizational commitment. At the same time, the product of the volunteers’ psychological capital, role identification, and perceived social support can have significantly negative predictive power on organizational commitment (β = 0.077, *p* < 0.01). These results showed that role identification and perceived social support could not only individually adjust the relation between volunteers’ psychological capital and organizational commitment, but also have a joint mediating effect on it. Therefore, both Hypothesis 3 and Hypothesis 4 were true as well.

**FIGURE 2:**
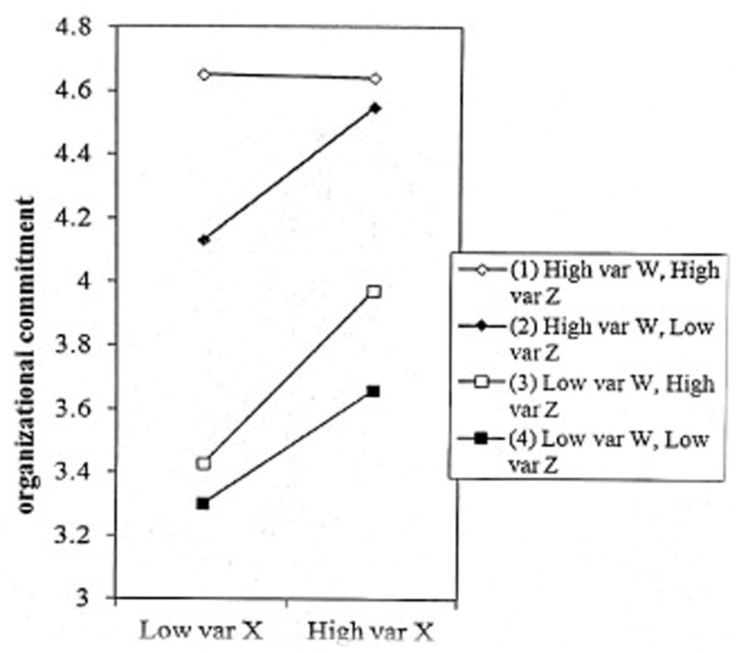
The joint moderated effect of role identification and perceived social support. “X” represents “Psychological Capital,” “W” represents “Role Identification,” and “Z” represents “Perceived Social Support.”

The present study calculated a simple slope and plotted a regulation effect diagram to better reveal the joint moderating trend of role identification and perceived social support in the relationship between the volunteers’ psychological capital and organizational commitment. As a result, compared with high role identification and high perceived social support [simple slopes (both high) = 0.033, t = 0.579, *p* > 0.05], it was more likely for volunteers’ psychological capital to induce organizational commitment under low role identification and low perceived social support [simple slopes (both low) = 0.279, t = 5.101, *p* < 0.01], high role identification and low perceived social support [simple slopes (high-low) = 0.238, t = 3.573, *p* < 0.01] and low role identification and high perceived social support [simple slopes (low-high) = 0.351, t = 3.893, *p* < 0.01]. To be specific, volunteers with low role identification and low perceived social support, high role identification and low perceived social support and low role identification and high perceived social support had a stronger relationship on organizational commitment when they were high in psychological capital; while volunteers with high role identification and high perceived social support had a faster impact on organizational commitment when they were low in psychological capital (see Figure 2 and Table 3).

**TABLE 3:** The significance test of slope difference.

Simple slop	B	SE
(1) VS (2)	−0.205**	0.076
(1) VS (3)	−0.318*	0.126
(1) VS (4)	−0.246**	0.089
(2) VS (3)	−0.113	0.135
(2) VS (4)	−0.041	0.092
(3) VS (4)	0.072	0.097

*represents *p* < 0.05; **represents *p* < 0.01.

## Discussion

Correlation analyses showed that volunteers’ psychological capital had a significant positive correlation with role identification, volunteering and organizational commitment, consistent with former studies on the relationships between psychological capital and role identification (Min and Ming-Jie, 2017), organizational commitment (Green and Chalip, 2004; Edwards, 2005; Bogler and Somech, 2019), and volunteering (Myers, 2012; Min and Ming-Jie, 2017). This meant that the positive psychological quality and positive mind state of volunteers could not only stimulate the degree of individual commitment to the organization in voluntary service, but also enable the individuals to have a greater acceptance of the role played by themselves thus leading them to attain more social support which would then prompt the behavior associated with continuing in the role (Grube and Piliavin, 2000). Moreover, as a positive psychological resource, psychological capital had the effect of replenishing energy and stimulating motivation (Wu et al., 2013; Datu et al., 2016). Individuals with higher psychological capital had better emotional regulation and cognitive strategies. They could better mobilize their positive psychological potential and were more likely to help others (Luthans et al., 2007; Myers, 2012).

The mediating effect analysis found that volunteers’ psychological capital could not only directly predict volunteering, but that psychological capital also had an indirect impact on volunteering through the mediating effect of organizational commitment. The result of the present study, that volunteers’ psychological capital had a direct positive prediction on volunteering, was basically consistent with previous results (Min and Ming-Jie, 2017; Cheng et al., 2018). In addition, psychological capital emphasized that individuals should give full play to their positive initiative and inherent potential advantages (Larson and Luthans, 2006). Volunteers with higher levels of psychological capital could not only better complete their jobs, but also were likely to help other volunteers, protect the volunteer organization and social resources, and effectively carry out their volunteering tasks and relative missions (Kragh et al., 2016).

Voluntary organizational commitment refers to a kind of psychological relationship that exists between volunteers and the volunteer organization, and which may promote a sustainable and healthy development of the organization (Francesco and Chen, 2004). In other words, it is the psychological attachment of volunteers to their organizations and the gratis contributions of volunteers to volunteer organizations and other volunteers. A study also considers that volunteers place a very high value on the work they do for the organization, and that their organizational commitment is regard as a combination of affective and continuance commitment (Edwards, 2007). The present study found that the product of volunteers’ psychological capital, role identification, and perceived social support can significantly negatively predict organizational commitment. To be specific, volunteers with low role identification and low perceived social support, high role identification and low perceived social support, and low role identification and high perceived social support had a greater impact on the organizational commitment when they had a high level of psychological capital; whereas, volunteers with high role identification and high perceived social support had a lower impact on organizational commitment when they have a high level of psychological capital. Resource conservation theory considers that it is easy to disperse resources in the process of psychological resources superposition (Wilson et al., 2010). Moreover, according to social exchange theory (Roch et al., 2019), although a high level of perceived social support contributes to stimulating a high level of role identification, volunteers are likely to generate overflow effects and negative effects under the high level of volunteers’ perceived social support and role identification. These overflow and negative effects increase the reward pressure on volunteers. Thus, volunteers will proactively reduce the level of organizational commitment to quell or balance these psychological states.

According to the resource conservation theory, the volunteers with high role identification and low perceived social support, and low role identification and high perceived social support were not using up resources too much because they had overlapping psychological resources (Wilson et al., 2010). Meanwhile, according to role theory (Broderick, 1998; Polzer, 2015) and social support theory (Lakey and Cohen, 2000), role identification and perceived social support are effective predictors of multiple behaviors. Therefore, even individuals with low perceived social support or low role identification may still be strongly committed to their organizations, which leads to more volunteering in turn. For the volunteers with low role identification and low perceived social support, their resources were neither depleted nor dispersed due to the high return required or the changing environment. Therefore, volunteers with low role identification and low perceived social support had a greater impact than high role identification and high perceived social support on organizational commitment when they had a high level of psychological capital.

As it turns out, the study also shows that volunteers’ perceived social support and role identification have a marginal effect on regulating psychological capital’s influence on organizational commitment. It reminds us to promote volunteers’ self-efficacy, resilience, optimism, and hope, to continuously strengthen the psychological relationship between volunteers and volunteer organizations. These factors can promote the sustainable and healthy development of volunteer organizations and increase the likelihood that volunteers will return after they first enter a volunteer organization, and that they will return when their role identification is not yet high and social support systems are not yet established. At the same time, in the comparatively mature volunteer organizations with higher levels of volunteers’ role identification and social support (Kramer, 1994; Griffin et al., 2000; Larson, 2017), more attention needs to be given to the balance between the psychological pressure and voluntary targets of volunteers, guiding volunteers to profoundly understand their advantages and disadvantages, and having a reasonable and effective evaluations of their targets. In the meantime, more matching voluntary services should be assigned to volunteers in accordance with their advantages and disadvantages, and supervisors should promote psychological balance and increase volunteering by adding more successful experiences to reduce psychological pressure.

### Theoretical Implications

There are at least three reasons why the present study is theoretically important. Firstly, the present study reveals how psychological capital influences volunteering. Previous research on volunteering has mostly focused on cultural capital, social capital, and human capital at home and abroad, yet research on psychological capital with respect to volunteering has not been done until now. Therefore, our study expands the perspective of research on the mechanism of sustainable development of volunteering.

Secondly, the present study enriches the research of psychological capital. Previous researches on psychological capital mainly focused on college students, teachers, employes, and researchers. Meanwhile, our study focused on volunteers, which helped to fill a gap in psychological capital research.

Thirdly, the present study has explored the “black box” of volunteers’ psychological capital on volunteering and its mechanism through integrated research. In addition, our study integrated the resource conservation theory and the main effect model of organizational commitment to reveal how and when volunteers’ psychological capital affects volunteering. These findings have not been reported in previous studies, and significantly enrich the research on volunteering (Benson et al., 2014).

### Practical Implications

From a practical perspective, our study takes volunteers served for the community as the research object to explore the impact of psychological capital on volunteering. Previous studies have designed training programs for volunteers from specific organizations including volunteers for the museum and the Olympic Games so that they can have the sustainability of volunteering (Green and Chalip, 2004; Edwards, 2005; Fornyth, 2010; Dickson et al., 2013, 2015; Darcy et al., 2014). Our research can provide some directions for the sustainable development of volunteering of those volunteers served for the community. For example, increasing the psychological capital of volunteers. Volunteer organizations can intervene psychological capital that help their volunteers learn mental regulation and be able to balance the psychological pressures and voluntary targets. For the volunteers who have joined voluntary organizations for a short time, they can be advised to constantly improve their psychological capital and strengthen their organizational commitment so as to promote their volunteering.

Furthermore, previous researches have shown that volunteer organizational commitment has a positive impact on voluntary behavior (Edwards, 2007; Matsuba et al., 2007). Our study also has shown that perceived social support and role identification has a joint mediation effect on the impact of psychological capital on organizational commitment. Therefore, voluntary organizations need to consider the role of volunteers in perceived social support and role identification when intervene their psychology to increase organizational commitment. For instance, the intervention is effective only when it is taken account of increasing perceived social support and reducing role identification or reducing perceived social support and increasing role identification, or simultaneously reducing perceived social support and role identification.

### Limitations and Future Research

The present study is not without limitations. Firstly, this study was cross-sectional, and although the time-lagged data reduces common method bias (Podsakoff et al., 2003), this research may restrict causal inferences. Thus, we encourage the use of experimental longitudinal designs to draw causal inferences in the future.

Secondly, volunteers aged 16-68 were included in this survey while those aged over 68 were not. Moreover, we looked at the volunteers as a whole and didn’t take account of the differences between young and old volunteers (such as differences in life experience, years of service, and perceptions and attitudes toward volunteer service) (Windsor et al., 2008). Therefore, it is necessary to strengthen the comparative study between young and old volunteers in future research.

Thirdly, more detailed measures were not involved because of the complexity of the model in this study. For example, we researched the mechanism of the composite psychological capital on volunteering but didn’t include how each individual dimension of psychological capital affected volunteering. Therefore, we encourage researchers to strengthen the study of the relationship between psychological capital and volunteering and reveal the role of organizational commitment, role identity, and social support.

Last but not least, there have been few studies on the psychological capital of volunteers at home and abroad, which may lead to a lack of in-depth analysis of the hypothesis and discussion in this study. The knowledge system of positive psychology and the study of positive organizational behavior have been enriched constantly. Many special advantages and virtues of individuals or groups have been put forward, and many factors have been found to meet the POB standard (Luthans et al., 2007). As a special group, the connotation and structure of the psychological capital of volunteers may have new characteristics. Therefore, we expect more researchers to pay attention to the psychological capital of volunteers and research it in the future.

## Conclusion

The present study suggest that volunteers’ psychological capital not only has a direct effect on volunteering, but that it also affects volunteering through the mediating role of organizational commitment. Besides, volunteers with low role identification and low perceived social support, high role identification and low perceived social support, and low role identification and high perceived social support commit to their organizations faster when they have a high level of psychological capital; whereas, volunteers with high role identification and high perceived social support commit to their organizations faster when they have a low level of psychological capital.

## Data Availability Statement

The datasets generated for this study are available on request to the corresponding author.

## Ethics Statement

This study was carried out in accordance with academic ethics guidelines, and the recommendations of the Committee of Zhuhai Campus of Zunyi Medical University, which also approved the study protocol. All subjects provided written informed consent in accordance with the Declaration of Helsinki.

## Author Contributions

LX designed, performed, and analyzed the research, and wrote the manuscript. YW revised the section of measures in manuscript and wrote responses to reviewer ZG. YW and JY searched literature. JZ analyzed and verified the data of this article.

## Conflict of Interest

The authors declare that the research was conducted in the absence of any commercial or financial relationships that could be construed as a potential conflict of interest.
